# A qualitative study exploring health seeking behaviour and cultural beliefs of mothers in paediatric malaria treatment decision-making process in Ile-Ife, South-West Nigeria

**DOI:** 10.1186/1475-2875-13-S1-P9

**Published:** 2014-09-22

**Authors:** Ibrahim S Bello, Satwinder Rehal

**Affiliations:** 1 Department of Public Health and Policy, University of Liverpool, Liverpool, UK

## Background

Reducing human infectivity of malaria parasite through early and effective treatment amongst under-five children is a major concern of WHO & MDGs 4 & 6. Getting mothers to bring their children to the nearest facility early for malaria treatment remains a major challenge.

## Aim

To explore health seeking behaviours and cultural attitudes of mothers of under five children suffering from malaria in Ile Ife using moderate interviews and providing recommendation to the Ministry of Health of Osun State to serve as knowledge basis for developing appropriate intervention.

## Materials and methods

The study employs a qualitative research with the aid of purposive sampling, 16 women who brought their under five children to hospital for malaria treatment were recruited. A moderate interview was used to explore the experiences of the mothers and the data generated was subjected to thematic content analysis.

## Results

Five themes emerged from the study. All respondents recognized fever as the major feature of malaria but their knowledge of what causes it, symptoms and sign was poor. They all perceived malaria as a simple disease for which mothers should be comfortable to apply home based remedies such as herbal concoctions. Religious beliefs did not prevent mothers from seeking hospital care but some are aware of mothers that rely on Holy water and prayer to treat malaria. Poverty is seen as an impediment to seeking treatment mostly by women in a polygamous relationship. Fear of use of injection to treat malaria in hospital coupled with perceived poor attitude of hospital staff was considered by some mothers.

## Conclusion

The study observed that mothers had poor knowledge of malaria; cultural beliefs in home remedies and poverty are issues that affect malaria outcome. Health education of the malaria life cycle, treatment and prevention is necessary for mothers as well as empowerment of women.

